# Promoting breastfeeding in women with gestational diabetes mellitus in high-income settings: an integrative review

**DOI:** 10.1186/s13006-023-00603-y

**Published:** 2024-01-18

**Authors:** Georgia Otter, Deborah Davis, Ella Kurz, Mary-Ellen Hooper, Alison Shield, Indira Samarawickrema, Sarah Spiller, Marjorie Atchan

**Affiliations:** 1 School of Nursing and Midwifery, University of Canberra, Bruce, Australia; 2 School of Health Science, University of Canberra, Bruce, Australia; 3 Health Care Consumer Association, Canberra, Australia

**Keywords:** Breastfeeding, Breast milk feeding, Gestational diabetes mellitus, Infant feeding, Barriers, Facilitators, Integrative review, High-income nations

## Abstract

**Background:**

Breastfeeding provides many short- and long-term health benefits for mothers and their infants and is a particularly relevant strategy for women who experience Gestational Diabetes Mellitus (GDM) during pregnancy. However, breastfeeding rates are generally lower amongst this group of women than the general population. This review’s objective is to identify the factors that influence breastfeeding by exploring the experiences and outcomes of women in in high-income health care contexts when there is a history of GDM in the corresponding pregnancy.

**Methods:**

A comprehensive search strategy explored the electronic databases Medline, CINAHL, Web of Science and Scopus for primary studies exploring breastfeeding practices for papers published between January 2011 and June 2023. All papers were screened independently by two researchers with included papers assessed using the Crowe Critical Appraisal tool. Findings were analysed using a narrative synthesis framework.

**Results:**

From an initial search result of 1037 papers, 16 papers representing five high-income nations were included in this review for analysis – the United States of America (*n* = 10), Australia (*n* = 3), Finland (*n* = 1), Norway (*n* = 1), and Israel (*n* = 1). Fifteen papers used a quantitative design, and one used a qualitative design. The total number of participants represented in the papers is 963,718 of which 812,052 had GDM and 151,666 did not. Women with an immediate history of GDM were as likely to initiate breastfeeding as those without it. However, they were more likely to have the first feed delayed, be offered supplementation, experience delayed lactogenesis II and or a perception of low supply. Women were less likely to exclusively breastfeed and more likely to completely wean earlier than the general population. Maternity care practices, maternal factors, family influences, and determinants of health were contextual and acted as either a facilitator or barrier for this group.

**Conclusion:**

Breastfeeding education and support need to be tailored to recognise the individual needs and challenges of women with a history of GDM. Interventions, including the introduction of commercial milk formula (CMF) may have an even greater impact and needs to be very carefully considered. Supportive strategies should encompass the immediate and extended family who are major sources of influence.

**Supplementary Information:**

The online version contains supplementary material available at 10.1186/s13006-023-00603-y.

## Background

Gestational diabetes mellitus (GDM) is a common medical condition reported during pregnancy, particularly in high-income healthcare settings [1, 2]. It is defined as glucose intolerance that emerges or is first recognised during pregnancy [3–5]. Unlike other types of diabetes, it is characterised by insulin resistance developed from placental hormonal release in which the maternal insulin response can no longer compensate for the insulin resistance, resulting in maternal hyperglycaemia [4].

The pooled standardised global prevalence of GDM is 14.0% with the highest prevalence of GDM across high-income nations [6]. GDM rates have dramatically increased due to several factors, such as increasing rates of obesity and maternal age, predisposition to GDM through a family history of type 2 diabetes mellitus (T2DM), or an ethnicity predisposed to developing GDM [1, 7, 8]. The increase has been further impacted by the introduction and wide global adoption of the World Health Organization (WHO) diagnostic guidelines in 2013, that have resulted in more women receiving a GDM diagnosis [1, 3, 9].

GDM increases the risk of adverse outcomes and long-term health complications for women and their infants [4, 8, 10]. Women with GDM have increased risks of complications during pregnancy including pre-eclampsia, hypertension, higher rates of birth trauma and birth interventions [2, 11–13]. Infants are also at increased risk of preterm birth, macrosomia, respiratory problems, and hypoglycaemia [14, 15]. Both women and their infants experience increased risks of longer-term impacts such as obesity and cardiometabolic disorders, with research revealing a tenfold increase in the risk of T2DM for mothers with a history of GDM [2, 16, 17], as well as a risk of women developing GDM in subsequent pregnancies [18]. There is also an increased risk of developing renal, ophthalmic or cardiovascular diseases for women and their infants [13, 15, 16, 19, 20]. The health care costs associated with GDM may also impact the health system. For example, in Australia, in 2019–20 GDM as a pregnancy episode was estimated to cost the health care system $63.6 million AUD, with hospital services accounting for 84% ($53.4 million AUD) [21]. This cost does not account for the goods and services required to manage any longer-term adverse health outcomes.

Despite the issues associated with GDM, it is known that the comorbidities and risks can be significantly reduced or managed with health behaviour changes, such as diet and physical exercise alongside monitoring blood glucose levels (BGL) [11, 22]. Some women may also require pharmacological management, using insulin or oral hypoglycaemic medications [23]. Optimal treatment during pregnancy reduces the incidence of GDM-related pregnancy complications including macrosomia and maternal hypertensive disorders [4]. In addition to health behaviour changes and antenatal interventions, studies have shown the importance of breastfeeding in improving longer-term outcomes for women with GDM and their infants [24, 25]. Aside from the benefits of exclusive breastfeeding to six months of life [26], further evidence suggests that being breastfed for longer periods of time reduces infants’ rates of obesity and diabetes in adulthood [27]. This finding has even more significance for women with a history of GDM and their infants, in reducing the longer-term risks associated with this condition.

Despite the known public health benefits of breastfeeding following a pregnancy complicated by GDM, current literature identifies issues of concern for mother-infant dyads with this complexity. Research demonstrates that women with GDM experience unmet care needs within current models of care [28, 29]. This is also of concern with the growing number of women experiencing GDM [2, 30]. A recent study suggested there were maternal and infant biological factors, provider practices, breastfeeding experiences and support plus cognitive and social factors contributing to the disparity in breastfeeding rates between women with a history of GDM and their non GDM counterparts [31]. A recent systematic review and meta-analysis also examined interventions for women with obesity and/or GDM to promote breastfeeding, finding that support increased initiation and duration for these women [32]. However, no reviews were found that explored the factors that positively influence or hinder the promotion and support of breastfeeding in women with GDM, to address this unmet need.

The objective of this review is to identify the factors that influence breastfeeding, as well as to explore the experiences and outcomes of women in in high-income health care contexts when there is a history of GDM in the corresponding pregnancy. Our review is unique in that it focusses on women with a recent GDM diagnosis only and does not include women with Type 1 diabetes mellitus (T1DM) or Type 2 diabetes mellitus (T2DM). The amount and availability of human and fiscal resources within a country may impact GDM care and subsequent breastfeeding practices, which limits the generalisability of findings, particularly to the Australian context. Consequently, this review has focussed on findings from high income nations, as defined by the World Bank, and who are presumed to have comparable health spending and burden, to increase confidence [33]. This review contributes to the field by integrating the best available evidence in the promotion of breastfeeding in women who have a recent history of GDM, to inform policy, practice and future research efforts.

## Methods

### Design

In addressing the objective of this review, studies relating to the breastfeeding experiences and outcomes of women with a recent history of GDM in high income healthcare settings, utilising quantitative, qualitative, or mixed methods methodology were included. We used Whittemore and Knafl’s integrative review methodology to guide the process: problem identification, literature search, data evaluation and extraction, data analysis, and presentation of results [34]. An integrative approach was taken to analysis and reporting whereby both quantitative and qualitative data are synthesised allowing for a comprehensive and holistic understanding of the topic. The research question guiding this review was: what are the breastfeeding experiences and outcomes of women when there is a history of GDM in the corresponding pregnancy? A research protocol was developed a-priori and published in the International Prospective Register of Systematic Reviews database (PROSPERO) (CRD42022292712).

### Search strategy and procedures

A search strategy was developed with the support of a specialist librarian with expertise in systematic reviews. The SPIDER (Sample, Phenomenon of Interest, Design, Evaluation, Research Type) framework was used to develop search terms [35] – see Table 1. The full search strategy is outlined in Supplementary Table 1.

**Table 1:** Sample search terms using the SPIDER framework

Sample	Phenomenon	Design	Evaluation	Research type
Postnatal/postpartum women/mothers with a history of GDM (No greater than 2 years postpartum, and no more than 2 years after the GDM affected pregnancy)	History of any/ “all” breastfeeding/breast milk feeding following a GDM affected pregnancy	Qualitative, quantitative, mixed methods	Experiences and Influencing factors (facilitators and barriers)	Primary research studies

The databases Web of Science, CINAHL, Scopus, and Medline were searched using the search terms. Searches were limited to publication date between January 2011 and June 2023 to reflect contemporary maternity care, and the filters ‘English’ language, and ‘human’ studies were applied where available.

### Inclusion and exclusion criteria

The following inclusion and exclusion criteria were applied to studies for this review. Studies were included if they: (1) focused on women with a diagnosis of, and treatment for, GDM during a recent pregnancy (participants were less than 2 years or postpartum from a pregnancy affected by GDM); (2) focused on ‘any’ form of breastfeeding or breast milk feeding; (3) highlighted or explored influencing factors or barriers of GDM on the woman’s breastfeeding experience; (4) used data collected from high-income nations – as defined by the World Bank [33]; (5) were published in the English language; and (6) were primary studies of a qualitative, quantitative or mixed methods design; and (7) were published in peer-reviewed journals from 2011 onwards. Studies were excluded if they did not meet all the inclusion criteria. Studies focussing on the antenatal, labour and birth rather than the postpartum period, or on women with T1DM or T2DM or other conditions associated with/or are present during pregnancy, were excluded. Additionally, studies that were not primary in nature such as reviews, abstracts, commentaries, editorials, or grey literature were excluded. The process was guided by the WHO operational definitions of breastfeeding [26]. High-income nations were identified using the World Bank criteria, with 81 nations holding this status in 2023 [33]. These nations have a Gross National Income (GNI) of $13,206 USD per capita or more and are associated with high-income healthcare.

**Fig. 1:**
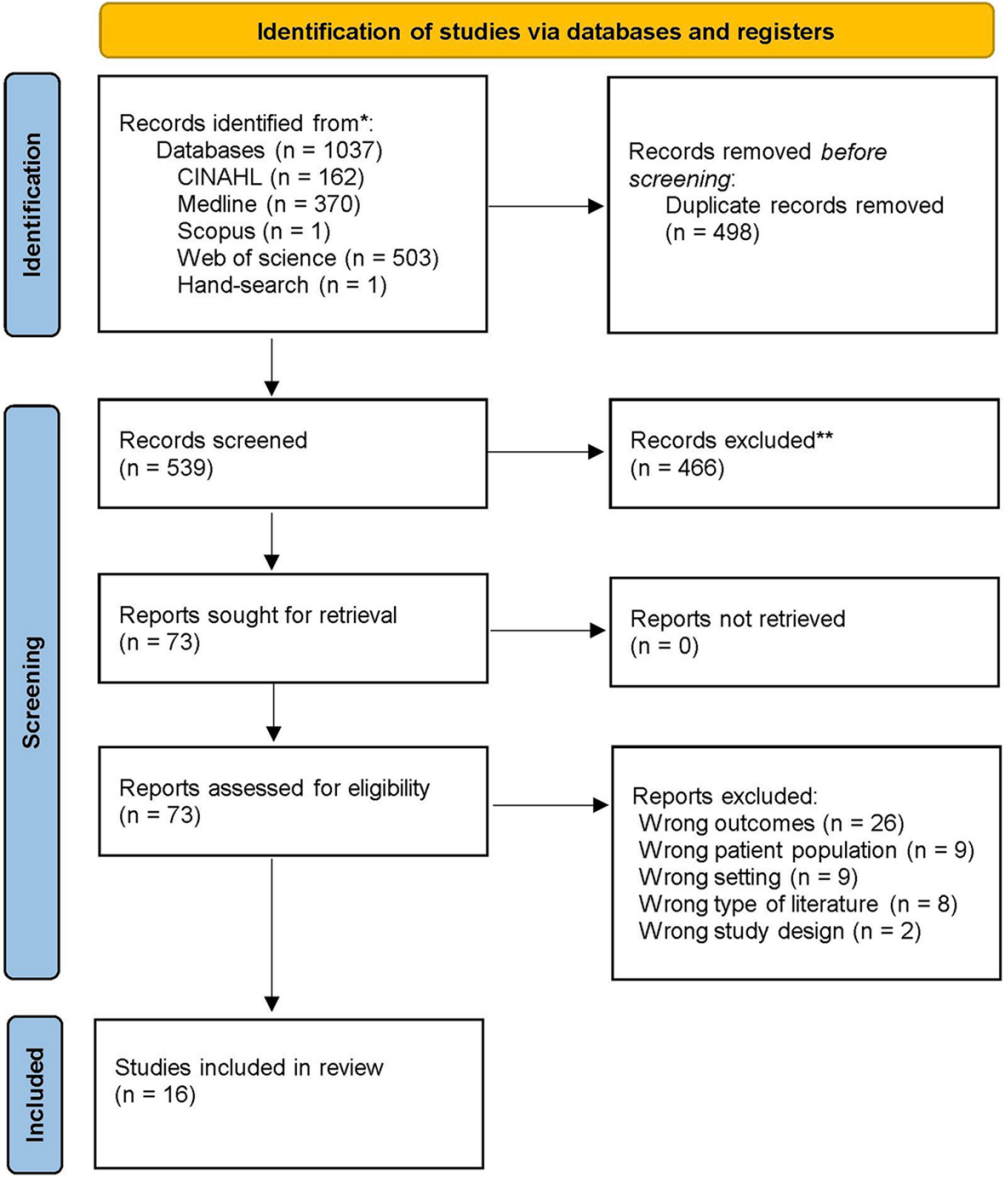
PRISMA flowchart

The selection of final papers for analysis followed the Preferred Reporting Items for Systematic Reviews and Meta-Analysis (PRISMA) flow chart as illustrated in Fig. 1 [36]. Papers were reviewed and managed using Covidence – an online systematic review tool, that allows for the screening, extraction, and analysis of data [37]. Following the search, all the identified citations were imported into Covidence. Duplicates were removed and the remaining papers were screened according to the inclusion and exclusion criteria [37]. All stages of the screening process were conducted independently by two authors, with any conflicts resolved through team discussion. GO and MEH conducted the search with the support of the specialist librarian and uploaded all citations to Covidence. Three authors conducted reviews based on title and abstract (GO, EK and MA). The full texts of selected papers were retrieved and assessed in detail against the inclusion criteria by three of the authors (GO, EK and MA).

### Quality assessment

The Crowe Critical Appraisal Tool (CCAT) v1.4 was utilised to critically appraise the full-text papers [38]. This tool allows for the appraisal for a variety of research designs and is used to appraise the design, sampling, data collection and ethical practices of research papers, with higher scores indicating high quality, rigorous, minimally-biased research [38]. There are eight domains in the CCAT, with details on the individual domains available in the CCAT User Guide [39]. Each domain was scored between 0–5, with 0 being lowest possible score and 5 the highest. Each full-text paper was appraised independently by two authors (MA, GO, MEH or EK).

### Data extraction and analysis

Data extraction and analysis was conducted by GO, MA and EK. Data from the included papers were manually extracted by two of the researchers, checked by a third and presented in tabular format to facilitate comparison and analysis across the studies – see Table 3. This table comprises data on the individual studies including the characteristics of the studies; positive and negative influences or factors to breastfeeding; and breastfeeding initiation and duration. Pooling of the data for meta-analysis was not conducted, given heterogeneity in the study designs, methods, and outcomes [40]. When heterogeneity exists, a narrative synthesis is considered appropriate to synthesise the data [40]. This approach allows for the findings to be integrated and allows for the exploration of similarities and differences among the studies [41]. This review adopted the narrative synthesis approach outlined by Popay et al.: developing a theory; developing a preliminary synthesis; exploring relationships in the data; and assessing the robustness of the synthesis [42]. The process of narrative synthesis was assisted with the use of NVivo Pro (version 12) software. In the process of data analysis, the findings of all papers were collated. Descriptive themes were constructed in a separate set of codes in NVivo and were discussed by the team and agreed on by consensus.

## Results

Database searches produced a total of 1037 publications. After duplicates were removed, 539 papers were screened by title and abstract; 466 papers were removed as their content did not meet the inclusion criteria. The full text of the remaining 73 papers were assessed for eligibility with 57 excluded at this stage (see Supplementary Table 2 for a list of excluded papers). Sixteen papers were included for analysis. Each of the 16 papers were assessed utilising the CCAT. Scores from the two independent appraisals were discussed and compared by the research team, with the average score for each paper outlined in Table 2. The independent scoring of each domain is also outlined in Table 2, providing a granular and transparent overview of the appraisal process. The total scores across the sixteen papers ranged from 68.75% to 87.5%, indicating satisfactory quality, with no papers excluded based on the CCAT score.

**Table 2:** Crowe Critical Appraisal Tool (CCAT) scores outlining the quality appraisal of included papers

First author & year	Reviewer	Preliminaries supporting text	Introduction supporting text	Design supporting text	Sampling supporting text	Data collection supporting text	Ethical matters supporting text	Results supporting text	Discussion supporting text	CCAT average score
Baerug 2018 [43]	R4	4	4	5	4	4	4	5	5	31.5/40
	R2	3	3	3	4	4	4	4	4	(78.75%)
Chamberlain 2017 [44]	R4	4	5	4	4	4	5	5	5	34.5/40
	R2	4	4	3	4	4	2	5	4	(86.25%)
Chertok 2016 [45]	R4	3	5	3	3	4	4	4	4	27.5/40
	R2	3	4	3	3	3	3	3	3	(68.75%)
Cordero 2013 [46]	R4	4	4	4	3	4	4	4	4	28/40
	R2	4	3	3	3	3	3	3	3	(70%)
Doughty 2018 [47]	R4	4	4	4	4	5	4	5	5	33.5/40
	R2	5	4	4	4	4	2	4	5	(83.75%)
Griffin 2021 [48]	R1	4	5	4	4	3	3	4	4	32/40
	R2	4	4	3	5	4	5	4	4	(80%)
Haile 2016 [49]	R3	4	4	4	3	4	3	5	4	28/40
	R2	3	3	3	5	3	2	3	3	(70%)
Jagiello 2015 [50]	R3	4	5	3	4	3	3	5	5	30.5/40
	R2	5	4	4	3	3	0	5	5	(76.25%)
Kachoria 2014 [51]	R3	5	4	4	5	5	5	5	5	34/40
	R2	4	4	3	4	4	3	4	4	(85%)
Laine 2021 [52]	R1	5	5	3	4	4	0	5	5	31.5/40
	R2	4	4	4	4	4	4	4	4	(78.75%)
Loewenberg Weisband 2017 [53]	R4	4	5	3	4	5	4	5	4	35/40
	R2	5	5	3	5	4	4	5	5	(87.5%)
Longmore 2020 [54]	R3	5	5	4	4	4	5	5	4	33.5/40
	R2	4	4	4	4	4	4	4	4	(83.75%)
Morrison 2015 [55]	R3	5	3	3	4	4	4	5	4	29.5/40
	R1	4	3	3	3	3	3	4	4	(73.75%)
Oza-Frank 2016 [56]	R3	4	4	3	4	4	3	4	5	29.5/40
	R2	3	4	4	3	4	2	4	4	(73.75%)
Oza-Frank 2017 [57]	R3	4	4	3	5	3	3	4	4	30.5/40
	R2	5	4	4	3	3	4	4	4	(76.25%)
Stuebe 2016 [58]	R1	5	4	4	4	5	4	5	4	34.5/40
	R2	4	4	5	4	5	3	5	4	(86.25%)

A total of sixteen papers were included in the review representing five high-income nations – the United States of America (*n* = 10), Australia (*n* = 3), Finland (*n* = 1), Norway (*n* = 1), and Israel (*n* = 1). The total number of participants represented in the papers is 963,718 of which 812,052 had a history of GDM and 151,666 did not. Fifteen papers used a quantitative design, and one used a qualitative design. The fifteen papers using a quantitative study design included: eleven cohort studies [43, 44, 46, 48, 49, 51–54, 56, 57]; two papers with both a cross-sectional and a cohort design [47, 55]; one prospective case control study [45]; and one randomised control trial [58]. Three papers used the US Infant Feeding Practices Study II data set [47, 49, 53] however, they reported on different aspects of it. Data collection methods varied between studies and included hospital records, birth registers, national statistics, and surveys. The single qualitative paper involved a phenomenological approach [50]. A summary of data extracted from the included papers is found in Table 3.

**Table 3:** Summary of included studies

Citation /title / location	Aim	Study design / sample	Data sources	Outcomes assessed / findings	Limitations	CCAT score
(Baerug et al., 2018) [43] Recent gestational diabetes was associated with mothers stopping predominant breastfeeding earlier in a multi-ethnic population Norway	To assess the association between GDM and breastfeeding in a multi-ethnic population	Retrospective Cohort study 823 women enrolled in the study. After removal of participants not meeting study inclusion criteria, 616 women included in the study who gave birth between May 2008 and May 2010 • Women with GDM = 190 • Women without GDM = 426	Women examined at 15- and 28-weeks’ gestation, and at 14 weeks’ postpartum Maternal data • Collected at the time of inclusion • Questionnaire completed by specially trained and certified midwives Birth data • Collected from hospital records Breastfeeding data Retrospective questionnaire covering the period since birth	á incidence of mothers with a history GDM ceasing breastfeeding earlier than mothers without a history GDM • aHR 1.33, 95% CI, 1.01—1.77, *p* < 0.05 Breastfeeding initiated by 99% of mother’s both with and without a history of GDM. Breastfeeding rates during the postpartum period: • End of week 1 postpartum: 86% of mothers with a history of GDM, versus, 91% of mothers without a history of GDM (*p* = 0.07) • End of week 2 postpartum: 78% of mothers with a history of GDM, versus, 88% of mothers without a history of GDM (*p* < 0.01) End of week 12 postpartum: 56% of mothers with a history of GDM, versus, 67% of mothers without a history of GDM (p = 0.02)	• Heterogeneity within broad ethnic groups • The use of plain water not included in the questionnaire, preventing the ability to assess exclusive breastfeeding according to the WHO definition • No data on if the administration of formula milk given in the infant first week of life occurred before or after hospital discharge • Lack of the one-hour glucose value	31.5/40 (78.75%)
(Chamberlain et al., 2017) [44] Low rates of predominant breastfeeding in hospital after gestational diabetes, particularly among Indigenous women in Australia Australia	To investigate rates of ‘any’ and ‘predominant’ breastfeeding in hospital among Indigenous and non-Indigenous women with and without GDM	Retrospective Cohort study Secondary analysis of data obtained from the Queensland Perinatal Data Collection (PDC) 670 participants identified who were born from 01/01/2007 – 31/10/2010 to a mother diagnosed with GDM. After removal of participants not meeting study inclusion criteria, 618 participants included in this analysis • Indigenous infants = 214 • Non-Indigenous infants = 404 Subgroup of 365 infants with medical record review • Indigenous infants = 209 Non-Indigenous infants = 156	Maternal data • The Cairns Hospital Clinical Coding System Pregnancy, birth, and breastfeeding data Queensland Perinatal Data Collection	â incidence of women with a history GDM predominantly breastfeeding • OR 0.32, 95% CI 0.27–0.38, *p* < 0.0001 â rates of breastfeeding amongst Indigenous Australian women with a history of GDM compared with non-Indigenous women with a history of GDM • OR 0.78, 95% CI 0.70–0.88), ≤ 0.0001)	• Reporting and coding of infant data was variable, and significant changes occurred during the study period, precluding the use of data prior to 2007 • Data only illustrates the 24 h prior to discharge • Secondary analysis, with limited data on characteristics of women who didn’t breastfeed	34.5/40 (86.25%)
(Chertok & Sherby, 2016) [45] Breastfeeding self-efficacy of women with and without gestational diabetes Israel	To identify factors that may contribute to maternal self-confidence in breastfeeding based on diabetes status	Subset of larger prospective case control study Potential participants were identified by medical staff on a postnatal ward. Recruitment was attended by the research team. Participants included 67 women who gave birth in an Israeli hospital between January and June 2014 • Women with GDM = 32 • Women without GDM = 35	Maternal and breastfeeding data • Surveys administered using the Breastfeeding Self-Efficacy Scale-Short Form (BSES-SF) Infant, birth, and glucose data Collected as per hospital protocol Infant, birth and glucose data was collected as per hospital protocol	á proportion of women without a history of GDM breastfed early in the first half hour following birth, compared to women with a history of GDM (*p* = 0.016) • Mothers with a history of GDM: 29% • Mothers without a history of GDM: 58.8% á proportion of women with a history of GDM reported perceived delayed lactogenesis II compared with women without a history of GDM Fischer’s exact test, *p* = 0.029	• Lack of consistency among healthcare providers in following hospital protocol about providing formula milk supplementation to babies born to women with GDM • Small sample size • No operational definitions for breastfeeding	27.5/40 (68.75%)
(Cordero et al., 2013) [46] Breastfeeding initiation in women with gestational diabetes mellitus United States	To examine feeding practices and factors associated with BFI in women with GDM and their infants	Retrospective cohort study Participants included 303 women who gave birth between 2008 and 2011 who had GDM during their pregnancy • 176 women with GDM treated with diet • 127 women with GDM treated with diet and insulin or Glyburide®	Maternal and clinical data • Electronic medical records Women’s feeding preference • Recorded by Nurse on arrival to labour and delivery ward Infant feeding data Hard copy and electronic neonatal medical records	0% of women with a history of GDM, who intended to formula milk feed, initiated breastfeeding 27% of women with a history of GDM, with undecided feeding preferences, initiated breastfeeding 54% of women, regardless of feeding preference, initiated breastfeeding Breastfeeding initiation rates were similar amongst women with a history GDM regardless of their GDM treatment method • OR 9.87 CI 4.96–19.64 á breastfeeding initiation for mother with a history of GDM associated with: • Intention to breastfeed: OR 9.87 CI 4.96–19.64 • Higher education: OR 4.20 CI 2.33–7.56 â breastfeeding initiation for mothers with a history of GDM associated with: • African American race: OR 0.40 CI 0.22–0.73 • Smoking: OR 0.26 CI 0.14–0.49 • Obesity: OR 0.39 CI 0.22–0.68 • Admission to the neonatal intensive care unit: 81% of infants born to mother with a history of GDM, receiving well baby care post birth whose mothers intended to breastfeed, initiated breastfeeding 61% of infants born to mothers with a history of GDM, who were admitted to the neonatal intensive care, unit whose mothers intended to breastfeed, initiated breastfeeding	• Limitations inherent to retrospective studies • Minimal follow up regarding breastfeeding practices • Unable to ascertain how far in advance mothers’ intention to or not to breastfeed was made • No operational definitions for breastfeeding	28/40 (70%)
(Doughty et al., 2018) [47] Barriers to exclusive breastfeeding among women with gestational diabetes mellitus in the United States United States	Identify differences in breastfeeding related knowledge, attitudes, beliefs, and experiences between women with GDM and women without GDM	Cross-sectional and prospective cohort study Subset of the Infant Feeding Practices Study II (IFPS II) conducted between 2005 and 2007 4902 women enrolled. 3033 completed the first postnatal questionnaire. After removal of participants not meeting study inclusion criteria, 1733 mother infant dyads included in this cohort • Women with GDM = 195 • Women without GDM = 2815	Mailed questionnaires • 1 antenatal questionnaire • 10 postnatal questionnaires, administered at monthly intervals following birth Phone interview Around the time of infant’s birth	â likelihood of mothers with a history GDM saying breastfeeding is the best way to feed an infant compared to mothers without a history GDM • aOR = 0.62, 95% CI (0.46, 0.85) á likelihood of mother with a history GDM stating the fathers of their infants prefer formula milk feeding or mixed feeding compared to mothers without a history of GDM • aOR = 1.74, 95% CI (1.02, 2.97) á likelihood of mothers with a history of GDM to state their physician prefers formula milk compared to mothers without a history of GDM • aOR = 2.82, 95% CI (1.17, 6.79) â likelihood of mother with a history of GDM to report feeling comfortable breastfeeding Infront of female friends compared to mothers without a history of GDM • aOR = 0.70, 95% CI (0.50, 0.98) â likelihood of newborns born to mothers with a history of GDM to stay in their mothers’ hospital room compared to infants born to mothers without a history of GDM aOR = 0.55, 95% CI (0.36, 0.85)	• IFPS II data collected 10 to 12 years before study published • Possibility of misclassification of the exposure to a diagnosis of GDM. Diagnosis of GDM self-reported by mothers • Data on disease severity and treatment not available for this cohort • No operational definitions for breastfeeding	33.5/40 (83.75%)
(Griffin et al., 2021) [48] Lactation consultation by an International Board-Certified Lactation Consultant improves breastfeeding rates for mother with gestational diabetes mellitus United States	To determine if a postpartum IBCLC consultation during delivery hospitalisation improved any or exclusive breastfeeding rates at hospital discharge and 3 months postpartum in women with GDM and To determine if obstetrical providers’ acknowledgement of maternal feeding preference affect the rates of IBCLC consultation for patients	Retrospective, comparative, secondary analysis of a prospective cohort Research staff approached women to discuss participation between January 2016 and December 2016 600 women enrolled in primary study. After removal of participants not meeting study inclusion criteria, 517 women with GDM were included in this secondary analysis	Maternal and in hospital breastfeeding data • Participant completed baseline surveys in either English or Spanish • Electronic medical records • Chart review to collect data regarding in-patient IBCLC consultation 3 months postpartum data • Scripted telephone survey	á likelihood of mothers with a history of GDM reporting any breastfeeding at postpartum discharge amongst mothers who received IBCLC consultation compare with mothers who did not • aOR 4.87, 95% CI (2.67, 8.86) á likelihood of mothers with a history of GDM reporting any breastfeeding at 3 months postpartum amongst mothers who received IBCLC consultation compare with mothers who did not • aOR 5.39, 95% CI (2.61, 11.16) There was no difference in exclusive breastfeeding rates between mothers with a history of GDM who did and did not receive IBCLC consultation	• Data collected retrospectively as part of secondary analysis • Data lacking regarding prior breastfeeding experience, prenatal breastfeeding education, and reasons for breastfeeding cessation and supplementation • The reason for a mother not receiving a lactation consultation not routinely documented in medical records • Mothers of infants with lower Apgar scored and neonatal intensive care unit admissions were less likely to receive a IBCLC consultation • Differences in provider group practices in addressing prenatal breastfeeding education • Differences in provider group documentation challenged the assessment of specific practices regarding prenatal breastfeeding education • No operational definitions for breastfeeding	32/40 (80%)
(Haile et al., 2016) [49] Association between history of gestational diabetes and exclusive breastfeeding at hospital discharge United States	To examine the association between GDM and exclusive breastfeeding at hospital discharge	Retrospective cohort study Subset of the Infant Feeding Practices Study II (IFPS II) conducted between 2005 and 2007 4900 participants in the IFPS II. After removal of participants not meeting study inclusion criteria, 2038 women were included in this study • Women with GDM = 119 • Women without GDM = 1919	Mailed questionnaires • 1 antenatal questionnaire • 10 postnatal questionnaires, administered at monthly intervals following birth Phone interview Around the time of infant’s birth	GDM prevalence was 5.8% â likelihood of women with a history of GDM exclusively breastfeeding a hospital discharge compared to women without a history of GDM (*P* < .01) • Women with a history of GDM:62.2% • Women without a history of GDM: 75.4% â odds of exclusive breastfeeding among women with a history of GDM after adjustment for sociodemographic, behavioural, and anthropometric factors OR = 0.59, 95% CI, 0.39–0.92	• Cross-sectional study design, precluding conclusions regarding the casual role of history of GDM in the status of exclusive breastfeeding • Women agreed to participate in the IFPSII survey, making the results vulnerable to volunteer bias, selection bias, and recall bias • No operational definitions for breastfeeding	28/40 (70%)
(Jagiello & Azulay Chertok, 2015) [50] Women’s experiences with early breastfeeding after gestational diabetes United States	To explore the lived experience of early breastfeeding for postpartum women who had GDM in pregnancy	A qualitative phenomenological research design Potential participants identified by medical staff during hospital postpartum stay or at lactation clinic visits. Interested participants were put in contact with the research team A purposive sample of 27 women who had been diagnosed with GDM and who initiated breastfeeding between October 2013 and January 2014	Breastfeeding experience data • Focus groups: Audio recorded, transcribed, and noted taken by a member of the research team • Interviews: Audio recorded, transcribed, and noted taken by a member of the research team • Questions used as prompts to initiate conversation and to provide structure • Session’s audio recorded Maternal data • Participant completed surveys	• Breastfeeding challenges and breastfeeding support - 44% (*n* = 12) of women used formula to manage breastfeeding challenges • Milk supply challenges - Delayed lactogenesis II was reported by 41% (*n* = 11) of women - Perceived decreased supply reported by 44% (*n* = 12) of women • Concern of infant health - 33% (*n* = 9) infants experienced complications including (15% (*n* = 4) with hypoglycaemia and 7% (n = 2) with jaundice)	• Small study sample size, limiting the transferability of the study findings • No operational definitions for breastfeeding	30.5/40 (76.25%)
(Kachoria & Oza-Frank, 2014) [51] Factors associated with breastfeeding at discharge differ by maternal diabetes type United States	To investigate the factors associated with breastfeeding initiation in mothers with gestational and prepregnancy diabetes vs those without diabetes	Retrospective cohort study 875,988 births in Ohio between 2006 and 2011. After removal of births not meeting the study inclusion criteria, 792,730 were used in this analysis	Maternal, infant, and breastfeeding data Birth certificates	The association of maternal and infant characteristics including maternal prepregnancy weight, maternal age, maternal race, prepregnancy care, county type, and infants gestational age on breastfeeding initiation, varied by maternal diabetes status Overweight mothers with a history of GDM were equally likely to breastfeed compared with mothers of normal weight, with a history of GDM • OR 0.95; 95% CI 0.87, 1.03 â likelihood of mothers with a history of GDM, from Appalachian countries, to breastfeed, compared to mothers with a history of GDM from suburban countries • OR 0.7; 95% CI 0.7, 0.8 â likelihood of mothers with a history of GDM who receive inadequate care to breastfeed, compared to mothers without a history of GDM • OR 0.8; 95% CI 0.7, 1.0	• Accuracy of birth certificate data not well researched in Ohio • Method of GDM diagnosis unknown • Limited information available regarding maternal complications of pregnancy and delivery • Lacks specific data about breastfeeding practices • No operational definitions for breastfeeding	34/40 (85%)
(Laine et al., 2021) [52] Impact of gestational diabetes mellitus on the duration of breastfeeding in primiparous women: an observational cohort study Finland	To evaluate in primiparous women whether GDM had an influence on the duration of breastfeeding, and further, to evaluate the factors that influenced on the duration of breastfeeding	Observational cohort study Participants included 1089 women who gave birth between 2009 and 2015 • Women with GDM = 155 • Women without GDM = 934	Maternal data • Finnish Medical Birth Register • Statistics Finland • Finnish Tax Administration • Social Insurance Institution Breastfeeding data • Health care records • Based on regular follow-up visits at public child welfare clinics Infant data Finnish Medical Birth Register	No differences observed in the duration of breastfeeding between women with a history of GDM and women without a history of GDM (*p* = 0.17) • Mothers with a history of GDM: 7.5 (SD 3.7) months • Mothers without a history of GDM: 7.9 (SD 3.5) months á duration of breastfeeding for male infants born to mothers with a history of GDM compared to female infants born to mothers with a history of GDM (maternal age, pre-pregnancy body mass index, marital status, educational attainment, duration of pregnancy, and smoking habits adjusted *p* = 0.042)	• Data only available on any breastfeeding, not separately on exclusively, predominant, and partial breastfeeding • Missing maternal data on women’s dietary and physical activity habits, as well as gestational weight gain • All women were Finnish, therefore the generalisability of the study observations is limited • No operational definitions for breastfeeding	31.5/40 (78.75%)
(Loewenberg Weisband et al., 2017) [53] Hospital supplementation differentially impacts the association between breastfeeding intention and duration among women with and without gestational diabetes mellitus history United States	To assess the associations between GDM and exclusive breastfeeding intentions, hospital supplementation, and breastfeeding duration, including whether hospital supplementation mediates the association between exclusive breastfeeding intention and breastfeeding duration	Retrospective cohort study Subset of the Infant Feeding Practices Study II (IFPS II) conducted between 2005 and 2007 4900 participants in the IFPS II. After removal of participants not meeting study inclusion criteria, 2299 women were included in this study • Women with GDM = 16 • Women without GDM = 2139	Mailed questionnaires • 1 antenatal questionnaire • 10 postnatal questionnaires, administered at monthly intervals following birth Phone interview Around the time of infant’s birth	â odds of intention to exclusively breastfeed associated with women with a history GDM • AOR 0.71; 95% CI, 0.51–0.99 á odds of hospital supplementation associated with both mothers with a history of GDM, and mothers without a history of GDM, who did not intend to exclusively breastfeed Mothers with GDM: AOR 3.52; 95% CI 1.44–8.57 Mothers without GDM: AOR 3.66; 95% CI 2.93–4.56 Mothers both with and without a history of GDM, who had exclusive breastfeeding intentions, breastfed for similar durations • Mothers with GDM: 22.3 weeks [95% CI (16.6–28.0); *p* < 0.001] Mothers without GDM: 20.7 weeks [95% CI (19.1–22.3); *p* < 0.001]	• IFPS II is not a nationally representative survey, limiting the generalisability of the findings • IFPS II data 10 + years old at time of study publication. Small sample size. 160 women with GDM, of whom, 127 women had information regarding supplementation • Intention to breastfeed assessed after GDM diagnosis, possibly influencing the woman’s intention to breastfeed • No operational definitions for breastfeeding	35/40 (87.5%)
(Longmore et al., 2020) [54] Associations of gestational diabetes and type 2 diabetes during pregnancy with breastfeeding at hospital discharge and up to 6 months: the PANDORA study Australia	To evaluate the association of hyperglycaemia, including type 2 diabetes, with breastfeeding outcomes	Longitudinal cohort study Cohort derived from 1170 participants in the Pregnancy and Neonatal Diabetes Outcomes in Remote Australia (PANDORA) cohort who gave birth between November 2011 – February 2015. After removal of participants not meeting study inclusion criteria, 1050 mother-infant dyads included in this study • Indigenous participants = 495 • Non-Indigenous participants = 555 • Women with GDM = 684 • Women without GDM = 222	Maternal data • PANDORA study • Medical records • Self-reported data • Australian Bureau of Statistics Infant data • Medical records Breastfeeding data • Direct questions attended by research team • Telephone or email survey attended by a member of the research team • Electronic medical records	Among women with a history of GDM, the proportions of those exclusively breastfeeding at hospital discharge was similar among both Indigenous and non-Indigenous Australian women • Indigenous women: 77% • Non-Indigenous women: 75% á likelihood of Indigenous Australian women with a history of GDM to be predominantly breastfeeding at 6 weeks postpartum, compared to non-Indigenous Australian women with a history of GDM. (*p* < 0.001) • Indigenous women: 81% • Non-Indigenous women: 57% á likelihood of Indigenous Australian women with a history of GDM to be predominantly breastfeeding at 6 months postpartum, compared to non-Indigenous women with a history of GDM. (*p* < 0.001) • Indigenous women: 68% • Non-Indigenous women: 46% Indigenous and non-Indigenous Australian women with a history of GDM were as likely to achieve predominant breastfeeding or exclusive breastfeeding at 6 weeks or 6 months as women without a history of GDM	• Loss of follow up at 6 weeks and 6 months may have affected the representativeness of the cohort • Sample size was not calculated for the purpose of addressing differences in breastfeeding outcomes • Potential for reporting bias • Women with diabetes may be more likely to report breastfeeding especially if they received information on potential benefits • Observational study, unmeasured confounding may influence findings	33.5/40 (83.75%)
(Morrison et al., 2015) [55] Factors associated with early cessation of breastfeeding in women with gestational diabetes mellitus Australia	To determine factors associated with early cessation of breastfeeding (< 3 months) in women with recent GDM	Cross-sectional online survey Cohort derived from 15,817 women registered with the National Diabetes Service Scheme in 2010 After removal of women not meeting study inclusion criteria, invitations were sent to 5057 women. Of the women invited, 738 women consented to participate. After further removal of women who did not meet the studies inclusion criteria, 729 eligible responses were included in this study	Maternal, infant, and breastfeeding data • Self-administered online questionnaire	97% of women with a history of GDM reported ever breastfeeding 19% of women with a history of GDM reported breastfeeding for 3 months Cessation of breastfeeding ≤ 3 months among women with a history of GDM associated with: • Breastfeeding problems at home: aOR 8.01, 95% CI 4.57, 14.05 • Return to work prior to three months: OR 3.39, 95% CI 1.53, 7.55 • Inadequate breastfeeding support: OR 1.88, 95% CI 1.10, 3.22 • Caesarean delivery: OR 1.70, 95% CI 1.04, 2.76 • Low socioeconomic status: (SEIFA 1 unit increase) OR 0.89, 95% CI 0.81, 0.97 • BMI (2 unit increase) OR 1.08, 95% CI 1.01, 1.57 Being married or de facto, was a protective factor against early cessation of breastfeeding for women with a history of GDM • OR 0.14, 95% CI 0.03, 0.62	• 15% response rate. 738 women completed the survey • Indigenous Australian women underrepresented • Potential for bias towards women interested in breastfeeding being more likely to respond to the survey • Women in the study were self-selected, highly educated, and differed somewhat from other Australian women with GDM, therefore, this study was unable to accurately estimate breastfeeding rates or make comparisons with GDM breastfeeding data reported elsewhere	29.5/40 (73.75%)
(Oza-Frank et al., 2016) [56] Early lactation and infant feeding practices differ by maternal gestational diabetes history United States	To examine breastfeeding practices through 12 months postpartum by GDM history	Retrospective Survey Design Population derived from the Mom2Moms (M2M) study. 1244 eligible women were identified who gave birth in 2011. After removal of women not meeting study inclusion criteria, surveys were mailed to the 813 eligible women. 501 completed surveys were returned. After further removal of women who did not meet the studies inclusion criteria, the final cohort included 432 women • Women with GDM = 34 • Women without GDM = 398	Maternal and infant data • Maternal medical records • Self-administered questionnaires Maternal lactation and infant feeding data • Self-administered questionnaire	Women with a history of GDM initiated any breastfeeding as often as women without a history of GDM á likelihood of women with a history of GDM reporting the introduction of formula milk within the first 2 days of life, compared to women without a history of GDM (aOR 3.48; 95% CI, 1.47–8.26) • Women with a history of GDM: 79.4% • Women without a history of GDM: 53.8%, Women with a history of GDM initiated pumping 4 days earlier than women without a history of GDM (*P* < 0.05) No difference in the proportion of women reporting breastfeeding difficulty when comparing women with and without a history of GDM OR 2.08; 95% CI, 0.78–5.52	• The subset of women with GDM in this sample is small and results may not be generalisable to all women with GDM history • The survey excluded women who intended to exclusively bottle feed, indicating the sample is more representative of women who intended to engage un some breastfeeding • Compared with non-responders, the sample was more educated, younger, had private health insurance, were non-Hispanic white, and reported fewer children • Did not have information on level of glycaemic control among women with GDM • No operational definitions for breastfeeding	29.5/40 (73.75%)
(Oza-Frank & Gunderson, 2017) [57] In-hospital breastfeeding experiences among women with gestational diabetes United States	To determine changes in the prevalence of hospital breastfeeding experiences among women with GDM and women without diabetes. And, to determine whether GDM is associated with higher occurrence of experiencing baby-friendly hospital practices because of their known higher rates of breastfeeding difficulties	Retrospective Observational Cohort Study Cohort derived from the Pregnancy Risk Assessment Monitoring System (PRAMS) between 2004 – 2008 and 2009—2011. 163,627 possible participants were identified in the two cohorts. After removal of participants not meeting study inclusion criteria, 157,187 participants were included amongst the two cohorts • Women with GDM = 14,409 • Women without GDM = 142,778	Maternal, infant, and breastfeeding data: • Self-administered questionnaire	2004 – 2008 Cohort â likelihood of mothers with a history of GDM reporting their baby stayed in the same room, compared to mothers without a history of GDM (*p* < 0.0001) • Mothers with GDM: 73.6% • Mothers without GDM: 78.3% â likelihood of mothers with a history of GDM reporting breastfeeding in hospital, compared to mothers without a history of GDM (not including pumping) (*p* = 0.01) • Mothers with GDM: 73% • Mothers without GDM: 75.7% â likelihood of mothers with a history of GDM reporting breastfeeding in the first hour following birth, compared to women without a history of GDM (*p* < 0.0001) • Mothers with GDM: 55.7% • Mothers without GDM: 65.5% â likelihood of mothers with a history of GDM reporting hospital staff helping them with breastfeeding, compared to mothers without a history of GDM (*p* = 0.03) • Mothers with GDM: 80% • Mothers without GDM: 82.5% â likelihood of mothers with a history of GDM reporting feeding only breastmilk in the hospital, compared to mothers without a history of GDM (*p* < 0.0001) • Mothers with GDM: 41% • Mothers without GDM: 52.4% â likelihood of mothers with a history of GDM reporting feeding on demand, compared to mothers without a history of GDM (*p* = 0.04) • Mothers with GDM: 81.2% • Mothers without GDM: 83.6% 2009 -2011 Cohort â likelihood of mothers with a history of GDM reporting their baby stayed in the same room, compared to mothers without a history of GDM (*p* < 0.003) • Mothers with GDM: 77.8% • Mothers without GDM: 81.8% â likelihood of mothers with a history of GDM reporting breastfeeding in hospital, compared to mothers without a history of GDM (not including pumping) (*p* < 0.01) • Mothers with GDM: 88.2% • Mothers without GDM: 91.9% â likelihood of mothers with a history of GDM reporting breastfeeding in the first hour following birth, compared to women without a history of GDM (*p* < 0.0001) • Mothers with GDM: 60.2% • Mothers without GDM: 68.6% â likelihood of mothers with a history of GDM reporting feeding only breastmilk in the hospital, compared to mothers without a history of GDM (*p* < 0.0001) • Mothers with GDM: 39.4% • Mothers without GDM: 52.7% á likelihood of mothers with a history of GDM reporting receiving a pump, compared to mothers without a history of GDM (*p* < 0.001) • Mothers with GDM: 39.5% • Mothers without GDM: 31.8% á likelihood of mothers with a history of GDM reporting receiving a formula gift pack, compared to mothers without a history of GDM (*p* = 0.002) • Mothers with GDM: 71% Mothers without GDM: 68.9%	• The data included in this study was from 16 states and New York City and therefor may not be generalisable to the whole of the United States • Variation in breastfeeding practices between facilities • Survey did not include data on all 10 BFHI steps (steps 1, 2 and 10 not asked) • The observational study design could allow for the possibility of reverse causality • PRAMS has limited data on maternity leave and employment • Potential for reporting bias • Criteria for GDM diagnosis unknown • ~ 10% of respondents with missing data excluded • No operational definitions for breastfeeding	30.5/40 (76.25%)
(Stuebe et al., 2016) [58] A Cluster Randomized Trial of Tailored Breastfeeding Support for Women with Gestational Diabetes United States	That a breastfeeding support intervention integrated within a lifestyle intervention would increase duration of any and exclusive breastfeeding among women with GDM compared to usual care	Randomised controlled trial 215 women screened for the study. After removal of women not meeting study inclusion criteria, 100 women were eligible and enrolled in the trial. Recruitment from 29/06/2012 – 11/09/2014 • Intervention group: 50 women • Control group: 50 women	Maternal, infant, and breastfeeding data • Women completed study assessments at - 22 – 36 weeks’ gestation - 6 weeks postpartum - At the end of the intensive intervention - 4 months postpartum - 7 months postpartum • 10 months postpartum	á Likelihood of women in the experimental group to be breastfeeding, and to be breastfeeding exclusively throughout the follow up period, when compared to women in the control group á rates of any and exclusive breastfeeding at 4 weeks postpartum amongst women in the experimental group when compared to women in the control group • Any breastfeeding: 87%. SE 5.4% in the experimental group, versus 64%. SE 7.7% in the control group • Exclusive breastfeeding: 45%. SE 7.9%. in the experimental group, versus 23%. SE 6.4% in the control group â likelihood of formula milk introduction amongst women in the experimental group when compared to women in the control group • aHR 0.50, 95% CI 0.34–0.72	• Cluster randomisation may not balance the groups as efficiently as individual randomisation • Higher loss of follow up than anticipated • Low follow up rates diminished the study’s ability to measure the extent to which the intervention increased achievement of Health People 2020 targets, including 3 month exclusive breastfeeding rates and 6 moth any breastfeeding rates • Low follow up rate might bias results • Breastfeeding results were self reported, providing for the possibility of social desirability bias to affect results • No operational definitions for breastfeeding	34.5/40 (86.25%)

The findings from this review have been integrated and are presented under four broad themes, constructed during the analysis. The four broad themes are: breastfeeding outcomes, maternity care practices, maternal factors and family influences, and underlying determinants of health, and these findings are outlined in Table 4.

**Table 4:** Findings

First author & year	Breastfeeding outcomes	Maternity care practices	Maternal factors and family Influences	Determinants of health
Baerug 2018 [43]	Y	-	-	Y
Chamberlain 2017 [44]	Y	-	-	Y
Chertok 2016 [45]	-	-	Y	-
Cordero 2013 [46]	Y	Y	Y	Y
Doughty 2018 [47]	-	Y	Y	-
Griffin 2021 [48]	-	Y	-	-
Haile 2016 [49]	Y	-	Y	-
Jagiello 2015 [50]	-	Y	Y	-
Kachoria 2014 [51]	Y	-	Y	-
Laine 2021 [52]	Y	-	Y	Y
Loewenberg Weisband 2017 [53]	Y	-	-	-
Longmore 2020 [54]	Y	-	-	-
Morrison 2015 [55]	Y	Y	Y	Y
Oza-Frank 2016 [56]	-	Y	-	-
Oza-Frank 2017 [57]	Y	Y	-	-
Stuebe 2016 [58]	-	Y	-	-

- equals no data

### Breastfeeding outcomes

Ten quantitative studies reported on breastfeeding outcomes – initiation and duration [43, 44, 46, 49, 51–55, 57].

#### Initiation

Cordero et al. found the most significant predictor of breastfeeding initiation was intention of breastfeeding [46]. However, one study found women with a recent history of GDM were less likely to report breastfeeding in the first hour (aOR 0.83; 95% CI 0.73, 0.94); feeding on demand (0.86; 0.74, 0.99); and feeding only breast milk in the hospital (0.73; 0.65, 0.82) in comparison to women without GDM [57]. Two papers reported similar findings. Loewenberg Weisband et al. found women with GDM were had a lower likelihood of intending to exclusively breastfeed than women without GDM (aOR 0.71; 95% CI 0.51, 0.99) [53]. Similarly, Chamberlain et al. also reported that women with GDM were also less likely to exclusively breastfeed than women without GDM (OR 0.32; 95% CI 0.27, 0.38, *P* < 0.0001) [44].

Two studies found similar rates of breastfeeding initiation regardless of GDM status. Kachoria and Oza-Frank found predictors of breastfeeding initiation were mostly similar among mothers with GDM and those without [51]. Whilst Baerug et al. found that 99% of all women initiated breastfeeding regardless of GDM status [43]. However, after 12 weeks, only 56% of the mothers with GDM compared to 67% of the mothers without GDM predominantly breastfed (*p* = 0.02) [43].

#### Duration

Three studies reported that women with GDM were less likely to breastfeed on discharge. Haile et al. found that at hospital discharge, 62.2% among women with a recent history of GDM exclusively breastfed, compared to 75.4% of women without GDM (*P* < 0.01) [49]. Longmore et al. similarly found that 75% (OR 0.7; 95% CI 0.4, 1.3) of women with GDM breastfed on discharge compared to 84% of women without GDM [54]. Morrison et al. found that while 97% of women with a recent history of GDM had ‘ever’ breastfeed, only 19% had breastfed for 3 months (*p* = 0.001) [55]. Two studies found there was no difference in the duration of breastfeeding when comparing women with GDM and those without GDM. Loewenberg Weisband et al. found that regardless of GDM, breastfeeding duration was similar when comparing exclusive breastfeeding intentions and by hospital supplementations [53]. Laine et al. also reported no differences in the duration of breastfeeding when comparing women with GDM (7.5 months [SD 3.7]) and those without GDM (7.9 months [SD 3.7]) (*p* = 0.17) [52].

Across the papers there were variations in the findings in relation to initiation and duration of breastfeeding. Women with GDM may be just as likely to initiate breastfeeding as women without GDM, however, across the studies, it appeared women with GDM were more likely to report delays to breastfeeding in the first hour, were less likely to exclusively breastfeed or were more likely to cease breastfeeding than women without GDM.

### Maternity care practices

Eight quantitative studies and the qualitative study reported on maternity care practices which influenced breastfeeding outcomes, these are largely birth interventions and complications, supplementation with CMF, and education and support [44, 46–48, 50, 55–58].

#### Birth interventions and complications

Birth interventions and complications after a pregnancy affected by GDM were associated with an increased risk of maternal-infant separation. Doughty et al. found that for women with GDM, newborns were less likely to stay with them in their hospital room than women without GDM (aOR 0.55; 95% CI 0.36, 0.85) [47]. Risk factors for not initiating or breastfeeding less than 30 days included infants with a health problem or prematurity [46]. Morrison et al. [55] describe an association between caesarean birth and the cessation of breastfeeding before 3 months (OR 1.70; 95% CI 1.04, 2.76) [55]. Similarly, Chamberlain et al. [44] reported lower breastfeeding rates among women having a preterm infant or caesarean birth [44]. Maternal-infant separation following birth was reported to affect breastfeeding, milk supply and bonding as one woman explains:

“*They let me see him for just a second and then they said that he needed to go to the nursery for monitoring. . . I didn’t get him skin to skin for hours*” [50]

#### Supplementation with CMF

Several papers reported on the use of CMF for women with GDM. Oza-Frank et al. [56] reported an increasing trend of women with a recent history of GDM being offered CMF as a strategy to address any breastfeeding challenges [56]. They found women with a recent history of GDM were more likely to introduce commercial milk formula (CMF) within the first two days (79.4%), than women without GDM (53.8%) (*P* < 0.01; aOR 3.48; 95% CI 1.47, 8.26) [56]. Oza-Frank et al. found women with a recent history of GDM were more likely to receive a pump (OR 1.28; 95% CI 1.07, 1.53) and a CMF gift-pack (OR 1.17; 95% CI 1.03, 1.34) compared with women without GDM [57]. Women with GDM were more likely to report that their physicians prefer CMF (aOR 2.82; 95% CI 1.17, 6.79) [47]. Jagiello and Azulay Chertok reported that the rate of CMF use during hospital stays where there was no medical indication was 68.4% (*n* = 39) [50]. The indication for the use of CMF was neonatal hypoglycaemia, along with other medical conditions. However, hospital records showed that CMF was given to these infants, even though it was not medically indicated [50].

Jagiello and Azulay Chertok found some women felt under supported by their maternity care providers and felt encouraged to supplement with CMF [50]:

*“The nurses in the hospital insisted on giving formula. Now the baby is not satisfied with breastfeeding and I am not sure that I have enough milk so I start with breastfeeding and then give formula.”* [50]

*“I was pretty traumatized at day four when I went to the pediatrician and they threw some formula at me and said . . . put your baby on formula because you’re not giving him enough.”* [50]

#### Education and support

Consistent support and advice was described as important to promote breastfeeding. Stuebe et al. found women with a recent history of GDM who received specialised breastfeeding education were less likely to stop breastfeeding (aHR 0.40; 95% CI 0.21, 0.74), or to introduce CMF (aHR 0.50; 95% CI 0.34, 0.72), than women with a recent history of GDM who did not receive the specialised education [58]. Griffin et al. found that women who had received an International Board-Certified Lactation Consultant (IBCLC) consultation were more likely to report ‘any’ breastfeeding on discharge (aOR 4.87; 95% CI 2.67, 8.86) and at 3 months postpartum (aOR 5.39; 95% CI 2.61, 11.16), compared to women who did not receive this consultation [48]. Jagiello and Azulay Chertok report the support of lactation consultations being highly valued in providing education, strategies, and advice to provide reassurance and address breastfeeding challenges [50]. As one woman describes:

*“[She] changed everything for me. When she came . . . and my mom was like . . . she’s worth her weight in gold.”* [50]

Women with GDM are more likely to experience delayed contact with their infants and are more likely to be encouraged to supplement with CMF. However, with appropriate and timely education and support, women with GDM can experience successful breastfeeding outcomes.

### Maternal factors and family influences

Seven quantitative studies and the qualitative study reported on maternal factors and family influences on breastfeeding [45–47, 49–52, 55].

#### Maternal factors

Chertok and Sherby found a significantly greater proportion of women with a recent history of GDM reported perceived delayed lactogenesis II compared with women without DM (Fisher’s exact test, *p* = 0.029) [45]. Whilst Jagiello and Azulay Chertok revealed that 41% of women in their study reported delayed lactogenesis II and 44% reported perceived insufficient milk supply [50]. A perception of insufficient milk supply was described by women as frustrating and feeling as though they were depriving their infant of nourishment [50]. Concerns for the infant’s health following birth also influenced breastfeeding. Among the infants in their study, 33.3% (*n* = 9) had experienced complications including hypoglycaemia (14.8%, *n* = 4) [50].

One large quantitative study [51] retrospectively reported that mothers who were overweight with a history of GDM were as likely to breastfeed as women with GDM without overweight (OR 0.95; 95% CI 0.87, 1.03) [51]. However, a lack of specific data on breastfeeding practices reduces confidence in the findings. Similar findings were reported by Haile et al. [49] with no statistical difference found between women who had normal gestational weight gain and women who exceeded the recommended guidelines [49]. However, Haile et al. [49] found that women who had gestational weight gain below the Institute of Medicine guidelines, were less likely to exclusively breastfeed in comparison to women who experienced normal gestational weight gain (OR 0.62; 95% CI 0.45, 0.85) [49].

Conversely, two studies found a greater body mass index (BMI) was associated with breastfeeding outcomes that do not meet the well-documented WHO guidelines [59]. Morrison et al. found that a higher BMI (2 unit increased) was associated with cessation of breastfeeding at or before 3 months (OR 1.08; 95% CI 1.01, 1.57) [55]. Similarly, Laine et al. found women who breastfed for less than 6 months had a higher pre-pregnancy BMI than women who breastfed for 6 months or longer (*P* < 0.001 for linearity) [52]. Cordero et al. also reported that being overweight or severely obese increased the likelihood of not breastfeeding at 30 days. This finding was associated with smoking during pregnancy and having a caesarean section [46].

#### Family influences

Morrison et al. found that breastfeeding problems at home was association with cessation of breastfeeding at or before 3 months (aOR 8.01; 95% CI 4.57, 14.05); returning to work within the first three months (OR 3.39; 95% CI 1.53, 7.55), and women experiencing inadequate breastfeeding support (OR 1.88; 95% CI 1.10, 3.22) [55]. However, Morrison et al. reported being in a de facto relationship or married was a protective factor against the early cessation of breastfeeding (OR 0.14; 95% CI 0.03, 0.62) [55]. Partner, family, and friend support were cited as supportive resources [50]:

*“My husband’s awesome . . . he’s like, [you should breastfeed] because it’s healthier for him and it’s healthier for you.”* [50]

Jagiello and Azulay Chertok found some women were encouraged to terminate breastfeeding and/or supplement with formula following breastfeeding challenges such as delayed lactogenesis or decreased milk supply [50].

*“and people are . . . like you should just stop, you should just pump, you should just use formula, why are you doing this?”* [50]

Women with GDM are more likely to experience delayed lactogenesis II or perceived insufficient milk supply and more likely to experience breastfeeding challenges than women without GDM. However, breastfeeding success can be enhanced in women with a supportive network and encouragement.

### Determinants of health

It appears that there are several determinants of health influencing breastfeeding outcomes and creating an added barrier for women with a history of GDM [43, 44, 46, 52, 55].

There is an association between ethnicity and breastfeeding outcomes. Chamberlain et al. [44] found lower breastfeeding rates among women who were Indigenous (53%) compared with women who were not (60%) (OR 0.78; 95% CI 0.70, 0.88, *P* < 0.0001) [44]. Baerug et al. [43] found women who were of South Asian ethnicity ceased predominant breastfeeding earlier than women of Western European ethnicity (aHR 1.53; 95% CI 1.04, 2.25) [43]. There also appears to be an association between socioeconomic status and breastfeeding outcomes with Morrison et al. [55] finding that cessation of breastfeeding at three months or earlier was increased in women of low socioeconomic status (SEIFA 1 unit increase) (OR 0.89; 95% CI 0.81, 0.97) [55]. More broadly, Laine et al. [52] found women who breastfed for less than six months were more likely to be younger, less well educated, or smokers, than women who breastfed for six months or longer (*P* < 0.001 for linearity) [52]. Cordero et al. [46] also found several factors associated with breastfeeding initiation failure, including lower education, obesity, smoking, and in their study, African American ethnicity [46]. The findings recognise that determinants of health may impact breastfeeding for all women however most authors suggest that women with added vulnerabilities experience additional barriers.

## Discussion

This review investigated the breastfeeding experiences and outcomes of women in high-income health care contexts when there was a history of GDM in the corresponding pregnancy. It was anticipated that the experiences and outcomes reported in the studies could reveal factors influencing breastfeeding or breastmilk feeding in women with GDM. This review found that there were differences in breastfeeding outcomes between women with GDM and women without GDM. Maternity care practices, maternal factors and family influences, as well as underlying determinants of health contributed to lower rates of breastfeeding in women with GDM.

There are multiple factors influencing the intention, initiation, and duration of breastfeeding amongst all women, regardless of GDM status. However, this review finds women with a recent history of GDM are even less likely to breastfeed than women without GDM or were even more likely to cease breastfeeding earlier than women without GDM. These results may be partially explained by the increased risk of pregnancy and birth complications for women with a recent history of GDM [31, 32]. For example, a recent prospective cohort study of 378 women with GDM during their pregnancy reported a statistically significant increase in the incidence of shoulder dystocia and caesarean section birth, as well as an increased probability of foetal distress and preterm infants [60]. Intrapartum interventions are known to contribute to maternal exhaustion, infant metabolic maladaptation and/or separation with supplementation and less favourable breastfeeding outcomes [61], even without the added complexity of GDM and unsupportive hospital clinical practice guidelines. It is well documented that injudicious interference with the normal physiology of lactogenesis will delay its onset.

Maternal factors and family influences as well as determinants of health also influence breastfeeding outcomes, a common finding across different high-income nations in this review. For example, women with a history of GDM who had reported increased rates of partner and family support for the initiation and continuation of breastfeeding, had greater odds of reporting breastfeeding initiation and exclusive breastfeeding [62–65]. This finding shows there is a clear relationship between the two concepts. A 2018 review of effective strategies to support breastfeeding indicated support and education strategies or interventions may improve breastfeeding practices, particularly if involving other family members such as the women’s mother or spouse [59]. The same review found additional supports were required for women with known medical complexities, in vulnerable or marginalised populations, and lower socio-economic status. Our review shows that women with a history of GDM may have medical and social complexities that further heighten their risk of not breastfeeding, intensifying the need for additional supports. Knowing that longer and more exclusive breastfeeding is known to be a protective factor for developing T2DM in the long term [66], antenatal and postnatal education needs to target the woman’s immediate family on the supportive measures that can be undertaken to improve the potential for breastfeeding success and further reduce the potential for adverse health outcomes.

It was found that maternity care practices influence breastfeeding in women with a recent history of GDM at all stages of the breastfeeding journey. Health professionals need to provide evidence-based breastfeeding support that is sensitive and tailored to the woman’s unique needs [67]. Our review affirms this practice, for example finding that women with an immediate history of GDM demonstrated better breastfeeding outcomes with support and the use of a lactation consultant [48, 50, 58]. In contrast, our findings also showed this group were more likely to be exposed to non-evidence-based practices such as being given breast pumps and encouraged to use CMF [50, 56, 57], disrupting the women’s sense of confidence in her ability to breastfeed successfully, also known as breastfeeding self-efficacy, which is an important predictor of initiation and the duration of breastfeeding [68, 69]. The development of breastfeeding self-efficacy is most vulnerable during late pregnancy and during the first week postpartum, being highly susceptible to the type of experiences encountered [68]. Our review shows that women with a history of GDM are more likely to encounter negative breastfeeding experiences than those without this condition, hampering their self-efficacy development and impacting their breastfeeding practices [50, 56, 57]. Maternity care practices that increase the probability of early successful experiences are crucial to implement in this group as a further protective measure against the known long-term impacts [2, 12, 24, 25, 27].

At multiple levels of society the call to create an environment that produces polices and practice guidelines free from commercial influence and protecting the rights of all women to make infant feeding decisions that meet their goals is getting stronger [70]. In a meta-analysis of the outcomes for the *Baby Friendly Hospital Initiative* (BFHI), several interventions were recommended to enhance breastfeeding outcomes. Rollins et al. [71] reported increased rates of exclusive breastfeeding (49%) and increased rates of any breastfeeding (66%) when BFHI breastfeeding support interventions were implemented. These interventions, as outlined in the *Ten Steps to Successful Breastfeeding* include early skin-to-skin contact and breastfeeding support, protection of lactation if mothers are separated from their infant, offering breastmilk substitutes only where there are clearly defined medical reasons, rooming-in and ongoing community support [72, 73]. The incorporation of BFHI recommendations into hospital guidelines as a routine set of practices in many high-income nations is needed [72], and when evidence-based breastfeeding support is not utilised, barriers to breastfeeding success are created [74]. Such advocacy is warranted for all women. Our findings suggest that women with a history of GDM could benefit from supports to enhance breastfeeding, and that widespread implementation of the BFHI package of interventions would present as modifiable opportunities.

### Recommendations for policy and practice

Recommendations for the enhanced support of the breastfeeding initiation and duration for women with a recent history of GDM have been drawn from the findings discussed above, namely:

1. Implement the full package of BFHI interventions
2. Specifically tailor antenatal and postnatal breastfeeding support to the individual needs of women diagnosed with GDM during their pregnancy
3. Involve the woman’s partner and family in the initiation and support of breastfeeding

While these recommendations are applicable to all women, the known challenges faced by women with GDM that impact breastfeeding need to be acknowledged and accounted for in management plans to mitigate risk factors.

### Strengths and limitations

This systematic integrative review is the first of its kind to examine and synthesise the experiences and outcomes of breastfeeding in women with a history of GDM in high-income settings, providing insights into the positive and negative influences on breastfeeding for women in this context. Being solely focussed on GDM rather than a combination of GDM, T1DM and T2DM adds strength and confidence to the findings. The papers included in this review were high quality with a range of CCAT scores between 68.75% and 87.5%. The range of high-income nations from which the studies originated increases the ability to generalise the findings to the Australian context because similar maternity care systems, as well as GDM screening and diagnostic processes may be used.

A limitation of this review was the lack of diversity amongst the study designs included. Most included papers used a quantitative study design, limiting our understanding of women’s experiences of breastfeeding following a GDM pregnancy. It is recognised that by not including low- and middle-income nations, some important research findings may have been missed. Another limitation is that studies included in the review did not uniformly control for variables which are likely to affect the initiation and duration of breastfeeding, regardless of status, for example, mode of birth, prior breastfeeding experience, smoking, obesity, socio-economic status, educational status, GDM treated with diet or medication, birth in a baby-friendly hospital. Further limitations identified in each study are listed in Table 2 and include a lack of operational definitions of breastfeeding, loss of follow up past 6 weeks and 6 months postpartum, differences in the way data was collected during the study period, and variation of breastfeeding practices between healthcare facilities.

### Further research

This review highlights a paucity of existing research related to women’s experiences of breastfeeding with a history of GDM. Future research should aim to understand the experiences of breastfeeding mothers with a history of GDM, not only in high-income contexts but in low- and middle-income. Understanding the woman’s experience will generate additional information, which when combined with the quantitative findings of this review, will be highly beneficial for improving maternity breastfeeding practices for both this cohort of women, and all breastfeeding women.

## Conclusion

This integrative review found the rates of initiation and duration of breastfeeding, were lower amongst women with a history of GDM when compared to their non GDM counterparts. Maternity care practices, such as those recommended by the BFHI, are particularly important in facilitating breastfeeding for mothers with a recent history of GDM. Maternal factors and family influences identified amongst the cohorts, can act as both facilitators and barriers to breastfeeding. Underlying social determinants of health including socioeconomic status appear to have a greater effect on women with a history of GDM than the wider birthing population. Appropriate, evidence-based, and timely professional support is key to a positive breastfeeding experience for all women with a recent history of GDM. Breastfeeding education and support need to encompass the individual needs of women with GDM and should include the immediate and extended family as they are major sources of influence. Well prepared, and supported women will have the ability to handle any challenges in achieving their infant feeding goals.

### Supplementary Information

**Additional file 1: Supplementary Table 1.** Search strategy via database. **Supplementary Table 2.** Excluded papers.

## Data Availability

The datasets used and/or analysed during the current study are available from the corresponding author on reasonable request.

## Abbreviations

BFHI: Baby Friendly Hospital Initiative
BGL: Blood Glucose Levels
BMI: Body mass index
CMF: Commercial milk formula
CCAT: Crowe Critical Appraisal Tool
GDM: Gestational Diabetes Mellitus
IBCLC: International Board-Certified Lactation Consultant
PRISMA: Preferred Reporting Items for Systematic Reviews and Meta-Analysis
T1DM: Type 1 diabetes mellitus
T2DM: Type 2 diabetes mellitus
