# Supplementary material for: Promoting breastfeeding in women with gestational diabetes mellitus in high-income settings: an integrative review
Source: Int Breastfeed J. 2024 Jan 18;19:4. doi: 10.1186/s13006-023-00603-y (PMC10795405; doi:10.1186/s13006-023-00603-y)
Supplement: Supplementary file 1 — Additional file 1: Supplementary Table 1. Search strategy via database. Supplementary Table 2. Excluded papers. [file 13006_2023_603_MOESM1_ESM.docx]

**Supplementary Table 1: Search strategy via database**

Search completed 1^st^ July 2023

Limiters: Jan 2011 and June 2023 – English, human

CINAHL

| #1 | (women OR mother* OR postnatal-women* OR postnatal-mother* OR postpartum-women OR postpartum-mother* OR maternal OR childbearing-women OR childbearing-mother* OR new-mother* OR recent*-birth* OR recent*-deliver*) | 441,176 |
| --- | --- | --- |
| #2 | (gestational-diabetes-mellitus OR gestational-diabetes OR gdm OR diabetes-in-pregnancy OR pregnancy-induced-diabetes OR diet-controlled-gestational-diabetes-mellitus OR diet-controlled-gdm OR insulin-requir*-gestational-diabetes-mellitus OR insulin-requir*-gdm OR gestational-hyperglycaemia OR gestational-glucose-intolerance OR IRGDM) | 9,467 |
| #3 | (breastfe* OR brest-fe* OR breastfeed*-women OR breastfeed*-mothers OR exclusive*-breastfe* OR full*-breastfe* OR predominant*-breastfe* OR any-breastfe* OR direct-breastfe* OR indirect-breastfe* OR mixed-breastfe* OR mixed-feed* OR expressed-breastmilk-feed* OR infant-feed* OR compl*-feed* OR suppl*-feed* OR intention-to-breastfe* breastfeed*-journey OR breastfeed*-experienc* OR intention-to-breastfeed commenc*-breastfeed* OR continu*-breastfeed* OR lactat*) | 36,796 |
| #4 | (barrier* OR facilitate* OR challenge* OR success* OR constrain* OR difficult* OR interfer* OR obstruct* OR problem* OR restrain* OR restrict* OR enable* OR factor* OR obstactle* OR imped* OR help* OR experienc* OR encount* OR achiev* OR limit* OR hard OR tough OR try* OR test* OR inhibit* OR delay* OR hinder* OR imped* OR complicat* OR setback* OR aid* OR assist* OR support* OR issue OR caus* OR commenc* OR continu* OR interrupt* OR disrupt* OR discontinu* OR ceas* OR stop* OR start* OR interrupt*) | 3,282,077 |
| #5 | 1 AND 2 AND 3 AND 4 | 261 |
| #6 | 5 AND (human) | 162 |

Medline

| #1 | (women OR mother* OR postnatal-women* OR postnatal-mother* OR postpartum-women OR postpartum-mother* OR maternal OR childbearing-women OR childbearing-mother* OR new-mother* OR recent*-birth* OR recent*-deliver*) | 913,528 |
| --- | --- | --- |
| #2 | (gestational-diabetes-mellitus OR gestational-diabetes OR gdm OR diabetes-in-pregnancy OR pregnancy-induced-diabetes OR diet-controlled-gestational-diabetes-mellitus OR diet-controlled-gdm OR insulin-requir*-gestational-diabetes-mellitus OR insulin-requir*-gdm OR gestational-hyperglycaemia OR gestational-glucose-intolerance OR IRGDM) | 16957 |
| #3 | (breastfe* OR brest-fe* OR breastfeed*-women OR breastfeed*-mothers OR exclusive*-breastfe* OR full*-breastfe* OR predominant*-breastfe* OR any-breastfe* OR direct-breastfe* OR indirect-breastfe* OR mixed-breastfe* OR mixed-feed* OR expressed-breastmilk-feed* OR infant-feed* OR compl*-feed* OR suppl*-feed* OR intention-to-breastfe* breastfeed*-journey OR breastfeed*-experienc* OR intention-to-breastfeed commenc*-breastfeed* OR continu*-breastfeed* OR lactat*) | 117,941 |
| #4 | (barrier* OR facilitate* OR challenge* OR success* OR constrain* OR difficult* OR interfer* OR obstruct* OR problem* OR restrain* OR restrict* OR enable* OR factor* OR obstacle* OR imped* OR help* OR experienc* OR encount* OR achiev* OR limit* OR hard OR tough OR try* OR test* OR inhibit* OR delay* OR hinder* OR imped* OR complicat* OR setback* OR aid* OR assist* OR support* OR issue OR caus* OR commenc* OR continu* OR interrupt* OR disrupt* OR discontinu* OR ceas* OR stop* OR start* OR interrupt*) | 11,084,445 |
| #5 | 1 AND 2 AND 3 AND 4 | 456 |
| #6 | 5 AND (human) | 370 |

Web of science

| #1 | (women OR mother* OR postnatal-women* OR postnatal-mother* OR postpartum-women OR postpartum-mother* OR maternal OR childbearing-women OR childbearing-mother* OR new-mother* OR recent*-birth* OR recent*-deliver*) | 1,648,587 |
| --- | --- | --- |
| #2 | (gestational-diabetes-mellitus OR gestational-diabetes OR gdm OR diabetes-in-pregnancy OR pregnancy-induced-diabetes OR diet-controlled-gestational-diabetes-mellitus OR diet-controlled-gdm OR insulin-requir*-gestational-diabetes-mellitus OR insulin-requir*-gdm OR gestational-hyperglycaemia OR gestational-glucose-intolerance OR IRGDM) | 27,886 |
| #3 | (breastfe* OR brest-fe* OR breastfeed*-women OR breastfeed*-mothers OR exclusive*-breastfe* OR full*-breastfe* OR predominant*-breastfe* OR any-breastfe* OR direct-breastfe* OR indirect-breastfe* OR mixed-breastfe* OR mixed-feed* OR expressed-breastmilk-feed* OR infant-feed* OR compl*-feed* OR suppl*-feed* OR intention-to-breastfe* breastfeed*-journey OR breastfeed*-experienc* OR intention-to-breastfeed commenc*-breastfeed* OR continu*-breastfeed* OR lactat*) | 219,238 |
| #4 | (barrier* OR facilitate* OR challenge* OR success* OR constrain* OR difficult* OR interfer* OR obstruct* OR problem* OR restrain* OR restrict* OR enable* OR factor* OR obstacle* OR imped* OR help* OR experienc* OR encount* OR achiev* OR limit* OR hard OR tough OR try* OR test* OR inhibit* OR delay* OR hinder* OR imped* OR complicat* OR setback* OR aid* OR assist* OR support* OR issue OR caus* OR commenc* OR continu* OR interrupt* OR disrupt* OR discontinu* OR ceas* OR stop* OR start* OR interrupt*) | 28,941,318 |
| #5 | 1 AND 2 AND 3 AND 4 | 728 |
| #6 | 5 AND (human) | 503 |

Scopus

| #1 | (women OR mother* OR postnatal-women* OR postnatal-mother* OR postpartum-women OR postpartum-mother* OR maternal OR childbearing-women OR childbearing-mother* OR new-mother* OR recent*-birth* OR recent*-deliver*) | 4,088,921 |
| --- | --- | --- |
| #2 | (gestational-diabetes-mellitus OR gestational-diabetes OR gdm OR diabetes-in-pregnancy OR pregnancy-induced-diabetes OR diet-controlled-gestational-diabetes-mellitus OR diet-controlled-gdm OR insulin-requir*-gestational-diabetes-mellitus OR insulin-requir*-gdm OR gestational-hyperglycaemia OR gestational-glucose-intolerance OR IRGDM) | 22,567 |
| #3 | (breastfe* OR brest-fe* OR breastfeed*-women OR breastfeed*-mothers OR exclusive*-breastfe* OR full*-breastfe* OR predominant*-breastfe* OR any-breastfe* OR direct-breastfe* OR indirect-breastfe* OR mixed-breastfe* OR mixed-feed* OR expressed-breastmilk-feed* OR infant-feed* OR compl*-feed* OR suppl*-feed* OR intention-to-breastfe* breastfeed*-journey OR breastfeed*-experienc* OR intention-to-breastfeed commenc*-breastfeed* OR continu*-breastfeed* OR lactat*) | 224 |
| #4 | (barrier* OR facilitate* OR challenge* OR success* OR constrain* OR difficult* OR interfer* OR obstruct* OR problem* OR restrain* OR restrict* OR enable* OR factor* OR obstacle* OR imped* OR help* OR experienc* OR encount* OR achiev* OR limit* OR hard OR tough OR try* OR test* OR inhibit* OR delay* OR hinder* OR imped* OR complicat* OR setback* OR aid* OR assist* OR support* OR issue OR caus* OR commenc* OR continu* OR interrupt* OR disrupt* OR discontinu* OR ceas* OR stop* OR start* OR interrupt*) | 28,761,043 |
| #5 | 1 AND 2 AND 3 AND 4 | 1 |
| #6 | 5 AND (human) | 1 |

**Supplementary Table 2: Excluded Papers**

| **Citation** | **Title** | **Reason for Exclusion** |
| --- | --- | --- |
| (Alyousefi et al., 2022) | Predictors of Prenatal Breastfeeding Self-Efficacy in Expectant Mothers with Gestational Diabetes Mellitus. | Wrong setting |
| (Carmody et al., 2019) | In-Hospital Feeding Practices of Infants Born to Mothers With Gestational Diabetes Mellitus or Type 2 Diabetes Mellitus: Evaluating Policy Implementation Effectiveness | Wrong outcomes |
| (Casey et al., 2019) | Perspectives and experiences of collecting antenatal colostrum in women who have had diabetes during pregnancy: a North Queensland semi structured interview study | Wrong outcomes |
| (Chapman, 2014) | Risk factors for delayed lactogenesis among women with gestational diabetes mellitus | Wrong type of literature |
| (Cherubini et al, 2022) | Breastfeeding Rates and Related Factors at 1 Year Postpartum in Women With Gestational Diabetes Initially Recruited for a Diabetes Prevention Program. | Wrong setting |
| (Cordero et al., 2022) | Exclusive breastfeeding among women with type 1 and type 2 diabetes mellitus. | Wrong study design |
| (Cordero et al., 2014) | Breast-feeding Initiation in Women with Pregestational Diabetes Mellitus | Wrong patient population |
| (Cummins, 2022) | What do women with gestational diabetes want for breastfeeding support? A participatory action research study | Wrong patient population |
| (Doughty & Taylor, 2021) | Barriers and benefits to breastfeeding with gestational diabetes | Wrong type of literature |
| (Doughty et al., 2016) | Prenatal Breastfeeding Attitudes and Postpartum Breastfeeding Experiences among Women with Gestational Diabetes Mellitus as Compared to Nondiabetic Women | Wrong type of literature |
| (Doughty et al., 2015) | Breastfeeding Intentions and Practices among Women in the US With Gestational Diabetes Mellitus | Wrong patient population |
| (Fallon & Dunne, 2015) | Breastfeeding practices that support women with diabetes to breastfeed. | Wrong type of literature |
| (Fenger-Grøn et al., 2015) | Low breastfeeding rates and body mass index in Danish children of women with gestational diabetes mellitus | Wrong outcomes |
| (Finkelstein et al., 2013) | Breastfeeding in women with diabetes: Lower rates despite greater rewards. A population-based study | Wrong outcomes |
| (Forster et al., 2017) | Advising women with diabetes in pregnancy to express breastmilk in late pregnancy (Diabetes and Antenatal Milk Expressing [DAME]): a multicentre, unblinded, randomised controlled trial | Wrong outcomes |
| (Glavey & Fallon, 2022) | Supporting women with diabetes to breastfeed: use of antenatal breastmilk expression. | Wrong type of literature |
| (Gouveri et al., 2011) | Breastfeeding and diabetes. | Wrong outcomes |
| (Gunderson et al., 2011) | Study of Women, Infant Feeding, and Type 2 diabetes mellitus after GDM pregnancy (SWIFT), a prospective cohort study: methodology and design. | Wrong outcomes |
| (Haile et al., 2019) | Combined Influence of Gestational Diabetes and Gestational Weight Gain on Exclusive Breastfeeding | Wrong outcomes |
| (Hebert et al., 2022) | Gestational Diabetes and Breastfeeding Among Women of Different Races/Ethnicities: Evidence from the Pregnancy Risk Assessment Monitoring Surveys | Wrong outcomes |
| (Huaxuan et al., 2020) | Effects of breastfeeding education based on the self-efficacy theory on women with gestational diabetes mellitus: A CONSORT-compliant randomized controlled trial | Wrong setting |
| (Jirakittidul et al., 2019) | Prevalence and associated factors of breastfeeding in women with gestational diabetes in a University Hospital in Thailand | Wrong setting |
| (Johnsen et al., 2021) | Antenatal breastmilk expression for women with diabetes in pregnancy - a feasibility study | Wrong outcomes |
| (Kachoria & Oza-Frank, 2014) | Differences in Breastfeeding Initiation by Maternal Diabetes Status and Race, Ohio 2006-2011 | Wrong outcomes |
| (Kaikini & Hyrkas, 2014) | Mothers' Intentions to Breastfeed and Hospital Practices on Breastfeeding: A Longitudinal Study at 6 Months After Birth on Predictors of Breastfeeding in a Cohort of Mothers From a Large Northern New England Medical Center. | Wrong type of literature |
| (Kim et al., 2020) | Knowledge and Health Beliefs of Gestational Diabetes Mellitus Associated with Breastfeeding Intention Among Pregnant Women in Bangladesh | Wrong setting |
| (Kole-White et al., 2021) | Breastfeeding Success Among Women with Gestational Diabetes Managed by Diet Only Compared with Those Requiring Medications | Wrong outcomes |
| (Kortsmit et al., 2023) | Prepregnancy and Gestational Diabetes and Cessation of Breastfeeding <1 Week Postpartum, United States, 2016-2018 | Wrong outcomes |
| (Kozhimanni et al., 2014) | Medically Complex Pregnancies and Early Breastfeeding Behaviors: A Retrospective Analysis | Wrong population |
| (Lis-Kuberka & Orczyk-Pawiłowicz, 2021) | Polish Women Have Moderate Knowledge of Gestational Diabetes Mellitus and Breastfeeding Benefits | Wrong outcomes |
| (Martin et al., 2020) | Maternal pre-pregnancy body mass index, gestational weight gain and breastfeeding outcomes: a cross-sectional analysis. | Wrong patient population |
| (Matias et al., 2014) | Maternal prepregnancy obesity and insulin treatment during pregnancy are independently associated with delayed lactogenesis in women with recent gestational diabetes mellitus. | Wrong outcomes |
| (Matsunaga et al., 2021) | Breastfeeding support and barriers to women with gestational diabetes mellitus: a nationwide cross-sectional survey of hospitals in Japan. | Wrong patient population |
| (Melov et al., 2022) | The BLIiNG study - Breastfeeding length and intensity in gestational diabetes and metabolic effects in a subsequent pregnancy: A cohort study. | Wrong outcomes |
| (Min et al., 2023) | Gestational diabetes and breastfeeding-related pain as major contributors to early breastfeeding cessation | Wrong outcomes |
| (Moorhead et al., 2022) | 'Is there any point in me doing this?' Views and experiences of women in the Diabetes and Antenatal Milk Expressing (DAME) trial. | Wrong patient population |
| Nguyen et al., 2019) | Gestational diabetes mellitus reduces breastfeeding duration: a prospective cohort study | Wrong setting |
| (Oza-Frank & Bartley, et al., 2014) | Differences in Breastfeeding Initiation and Continuation by Maternal Diabetes Status | Wrong outcomes |
| (Oza-Frank et al., 2014) | Differences in breast-feeding initiation and continuation by maternal diabetes status | Wrong outcomes (duplication) |
| Oza-Frank, et al., 2015) | Differences in breast-feeding initiation and continuation by maternal diabetes status | Wrong outcomes |
| (Oza-Frank et al., 2017) | In-hospital breastfeeding experiences among women with gestational diabetes | Wrong type of literature |
| (Panaviene et al., 2019) | Factors Contributing to Non-Exclusive Breastfeeding in Primigravid Mothers | Wrong outcomes |
| (Pang et al., 2021) | The association of maternal gestational hyperglycemia with breastfeeding duration and markers of milk production | Wrong outcomes |
| (Park, et al., 2021) | Factors Associated with the Need for Breastfeeding Information Among Women with Gestational Diabetes Mellitus: A Cross-sectional Study | Wrong population |
| (Park et al., 2018) | Knowledge and health beliefs about gestational diabetes and healthy pregnancy's breastfeeding intention | Wrong outcomes |
| (Pinheiro et al., 2018) | Maternal pre-pregnancy overweight/obesity and gestational diabetes interaction on delayed breastfeeding initiation | Wrong outcomes |
| (Poorghasemian et al., 2022) | The Effect of Counseling on Breastfeeding Self-Efficacy (BSFE) in Women with Gestational Diabetes | Wrong outcomes |
| (Qian et al, 2023) | Decision-making process of breastfeeding behavior in mothers with gestational diabetes mellitus based on health belief model. | Wrong setting |
| (Rasmussen et al., 2020) | Factors associated with breastfeeding to 3 months postpartum among women with type 1 and type 2 diabetes mellitus: An exploratory study. | Wrong patient population |
| (Reinheimer et al., 2020) | Factors Associated With Breastfeeding Among Women With Gestational Diabetes | Wrong setting |
| (Shen et al., 2019) | Lactation intensity and duration to postpartum diabetes and prediabetes risk in women with gestational diabetes | Wrong outcomes |
| (Stevens et al., 2019) | Breastfeeding Initiation as Related to the Interaction of Race/Ethnicity and Maternal Diabetes | Wrong outcome |
| (Tanda et al., 2018) | Factors That Modify the Association of Maternal Postpartum Smoking and Exclusive Breastfeeding Rates | Wrong outcome |
| (Verd et al., 2016) | The Effects of Mild Gestational Hyperglycemia on Exclusive Breastfeeding Cessation | Wrong setting |
| (Wallenborn et al., 2017) | Breastfeeding after Gestational Diabetes: Does Perceived Benefits Mediate the Relationship? | Wrong outcomes |
| (Wang et al., 2020) | Exploring the breastfeeding knowledge level and its influencing factors of pregnant women with gestational diabetes mellitus | Wrong setting |
| (Youngwanichseth, 2013) | Factors related to exclusive breastfeeding among postpartum Thai women with a history of gestational diabetes mellitus | Wrong setting |
